# Supplementary material for: Targeting the Pentose Phosphate Pathway for SARS-CoV-2 Therapy
Source: Metabolites. 2021 Oct 13;11(10):699. doi: 10.3390/metabo11100699 (PMC8540749; doi:10.3390/metabo11100699)
Supplement: Supplementary file 1 [file metabolites-11-00699-s001.zip › metabolites-1372053-supplementary.pdf]

Figure S1

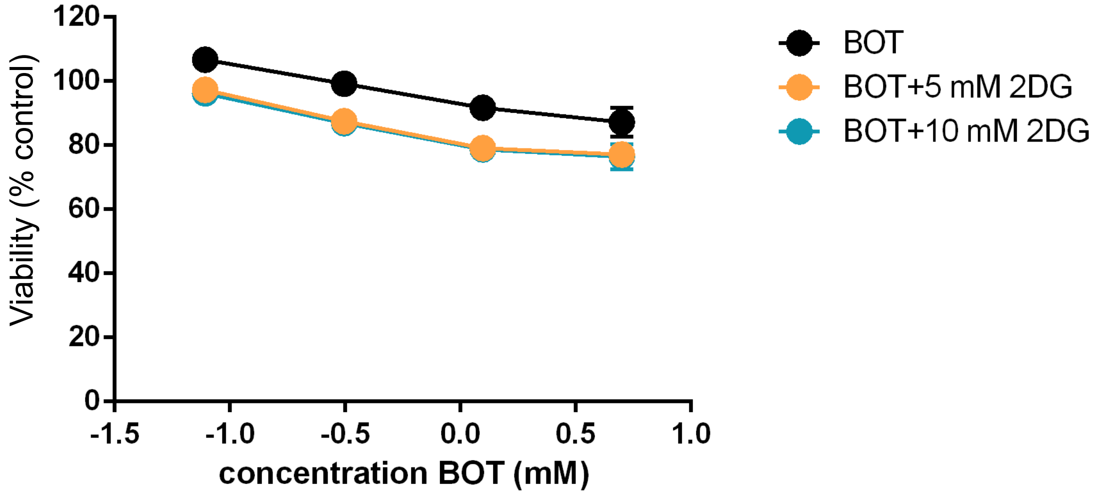

Figure S1. Effects of Benfooxythiamine (BOT) with or without 2-deoxy-D-glucose (2DG) on the viability of non-infected Caco-2 cells treated analogously to SARS-CoV-2-infected cells in Figure 4 as determined by MTT assay.
